# The Effect of Surface Entropy on the Heat of Non-Wetting Liquid Intrusion into Nanopores

**DOI:** 10.1021/acs.langmuir.1c00005

**Published:** 2021-04-12

**Authors:** Alexander R. Lowe, William S. Y. Wong, Nikolay Tsyrin, Mirosław A. Chorążewski, Abdelali Zaki, Monika Geppert-Rybczyńska, Victor Stoudenets, Antonio Tricoli, Abdessamad Faik, Yaroslav Grosu

**Affiliations:** † Institute of Chemistry, University of Silesia, Szkolna 9, 40-006 Katowice, Poland; ‡ Nanotechnology Research Laboratory, College of Engineering and Computer Science, The Australian National University, Canberra ACT 2601, Australia; § Laboratory of Thermomolecular Energetics, National Technical University of Ukraine “Igor Sikorsky Kyiv Polytechnic Institute”, Pr. Peremogy 37, 03056 Kyiv, Ukraine; ∥ Centre for Cooperative Research on Alternative Energies (CIC energiGUNE), Basque Research and Technology Alliance (BRTA), Alava Technology Park, Albert Einstein 48, 01510 Vitoria-Gasteiz, Spain; ⊥ Materials Science, Energy and Nano-engineering Department, University Mohammed VI Polytechnic, Lot 660, Hay Moulay Rachid, 43150 Ben Guerir, Morocco; △ Nanotechnology Research Laboratory, University of Sydney, 2006 New South Wales, Australia

## Abstract

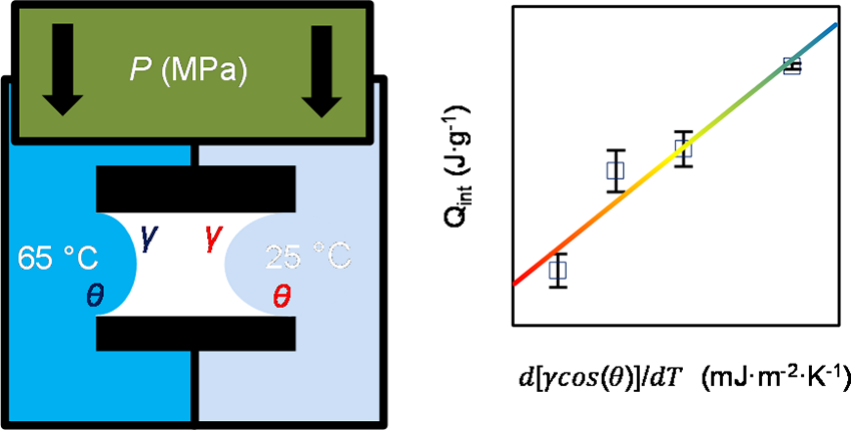

On-demand access to renewable and environmentally friendly energy sources is critical to address current and future energy needs. To achieve this, the development of new mechanisms of efficient thermal energy storage (TES) is important to improve the overall energy storage capacity. Demonstrated here is the ideal concept that the thermal effect of developing a solid–liquid interface between a non-wetting liquid and hydrophobic nanoporous material can store heat to supplement current TES technologies. The fundamental macroscopic property of a liquid’s surface entropy and its relationship to its solid surface are one of the keys to predict the magnitude of the thermal effect by the development of the liquid–solid interface in a nanoscale environment—driven through applied pressure. Demonstrated here is this correlation of these properties with the direct measurement of the thermal effect of non-wetting liquids intruding into hydrophobic nanoporous materials. It is shown that the model can resonably predict the heat of intrusion into rigid mesoporous silica and some microporous zeolite when the temperature dependence of the contact angle is applied. Conversely, intrusion into flexible microporous metal–organic frameworks requires further improvement. The reported results with further development have the potential to lead to the development of a new supplementary method and mechanim for TES.

## Introduction

The thermal effect (heat) of intrusion-extrusion is a fascinating phenomenon, which is generated when a non-wetting liquid is spread over a lyophobic nanoporous material’s surface.^1−12^ The phenomena of wetting and de-wetting and its related effects are relevant for various applications such as molecular springs,^1,4,7^ nanobumpers,^13−18^ porosimetry,^12^ column chromatography,^19,20^ reusable energy absorbers,^21,22^ and self-cleaning behavior^23−25^ and have been exploited for enhanced oil recovery.^26^ From a molecular perspective, the non-wetting liquid prefers to interact with molecules of its own kind (cohesion) rather than associate with those of the solid interface material (adhesion). Hence, bringing these substances closer together can be thought of as an energy-consuming process, which is required to overcome the associated energy barriers. An example case is the forced intrusion of water into various hydrophobic solids.^3,6,9,10,13^ The process is associated with the application of mechanical and thermal energy to the system.^5,7^ These stored mechanical (work) and thermal (heat) energies can then be released upon spontaneous extrusion, which gives rise to several applications in the field of energy storage,^11,27−30^ dissipation,^15,16,31,32^ and conversion.^8^ So far, the intrusion-extrusion process has been extensively investigated in terms of its mechanical aspects, while thermal effects remain poorly studied. This is mainly due to the lack of sophisticated high-pressure calorimetry techniques required for these studies.

At the time of writing, calorimetric studies for non-wetting liquid intrusion are scarce in the literature, with existing studies providing limited insights into the process of heat generation during intrusion and extrusion. Coiffard *et al.*(5) used scanning transitiometry (*PVT*-calorimetry), which simultaneously recorded both the mechanical and thermal properties of water intrusion into nanoporous solids of either grafted silica or hydrophobic silicates. It was observed that large pore mesoporous materials generated exothermal heat upon the intrusion of water into a 4 nm pore, while the smaller microporous samples were endothermic for a 0.3 nm pore.^5^ The extrusion properties of all the discussed systems were exothermic. Karbowiak *et al.*(1) did a similar study with water and silicalite-1 using a modified Setaram C-80 calorimeter and showed a strong endothermic thermal response to the first liquid intrusion cycle, with subsequent intrusion processes demonstrating significantly weaker thermal signals. This reduction was identified as the result of chemical degradation of the pore surface, which created hydrophilic silanol groups. These initial intrusion results were confirmed by Ievtushenko *et al.,*(10) who proposed a thermodynamic model to predict the thermal output during intrusion-extrusion.

Since then, the calorimetric studies of water intrusion-extrusion were extended to include metal–organic frameworks (MOFs), which consist of ZIF-8^7,9^ and Cu_2_(tebpz) (tebpz = 3,3′,5,5′-tetraethyl-4,4′-bipyrazolyl).^4^ The calorimetric experiments conducted on ZIF-8 showed both the heat of endothermic intrusion and the heat of exothermic extrusion, which were measured up to 25 J·g^–1^ at a temperature of 90 °C. The material also demonstrated endothermic interface development upon isobaric cooling, demonstrating a new mode of operation.^7^ Cu_2_(tebpz) was shown to be a very stable and near-perfect molecular spring capable of storing 7.9 J·g^–1^ of thermal energy at a similar temperature.^4^ In addition, the negligible hysteresis of this system enables it to efficiently store both mechanical and thermal forms of energy. All the aforementioned systems were investigated with water as the non-wetting liquid.

Cailliez *et al.*(6) studied how the pore geometry affects the thermal effect of water intrusion into a narrow hydrophobic environment. The team used a combination of experimental pressure–volume data for the intrusion of water into the pores and Grand Canonical Monte Carlo simulations in order to provide a thermodynamic description of the intrusion process as a first-order phase transition. They determined that the thermal effect can be either endothermic or exothermic depending on the pore size. Similar experimental results were observed by Karbowiak *et al.*(2) and Coiffard *et al.*(5) The change in thermal energy sign (positive/negative) is due to the number of water molecules exposed to the hydrophobic surface in the narrow pore. Consequently, this can also be called the heat of interface development. Laouir *et al.*(33) have expressed the idea of harnessing this energy to drive molecular machines/engines/motors as proposed by Eroshenko.^28,31,34,35^ The work of Laouir *et al.*(33) provides a complete thermodynamic analysis of the energy produced with respect to an ideal cycle of the film engine or “Stirling cycle”. While less efficient when compared to the ideal Carnot cycle, these ideal cycles have the potential to serve as the basis for driving molecular machines and by extension heat pumps, particularly when a compact solution is required. The description they provided link to the thermal effects of the fundamental properties of a liquid at the macroscopic scale, which includes the surface tension, γ, contact angle, θ (radian), surface entropy, dγ/d*T*, and temperature dependence of the contact angle, dθ/d*T* [(radian)·K^–1^]. These liquid properties are linked to the thermodynamic response of both thermal and mechanical energy. A more complex model by Borman *et al.*(36) used analytical methods with percolation theory as applied to randomly situated spheres. Tersely, the model identified that the exothermic and endothermic behavior of intrusion and extrusion is a combination of surface development, which is related to the surface energy and meniscus formation, which is associated with the temperature dependence of contact angle. Consequently, the model evaluated the effects of pore filling in a vacuum and the thermal energy development during this process.

The largest number of applications have been reported to coincide with the Laplace equation:
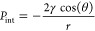
1which relates both the surface tension γ and contact angle θ of the liquid to the pressure needed for it to enter a pore with the radius, *r.* This is defined as the intrusion pressure *P*_int_. Understandably, this indicates that large surface tensions and contact angles greater than 90° will require proportionally larger pressures to induce intrusion. This also means that larger pores will require less pressure to force the liquid to enter it. Once the liquid begins to spread into the pore and create the solid–liquid interface, thermal energy is generated. This has been expressed by Laouir *et al.*(33) as the “modified Kelvin equation”, but it is best understood as the macroscopic Gibbs heat of the solid–liquid interface development-reduction:
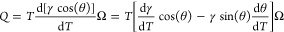
2where *Q* (J·g^–1^) is the heat of interface development-reduction, *T* is the temperature, and Ω (m^2^·g^–1^) is the specific surface area of a porous material. Here, interfacial interactions play a significant role and require a closer look regarding the effects of the dcos(θ)/d*T* and dγ/d*T*, with the evaluation of these properties as the justification for this work. It is understood that, in nanopores, the properties of the liquid can be sufficiently different compared to the bulk. However, understanding whether the heat of intrusion of a non-wetting liquid into nanopores can be predicted using its bulk properties of the θ, γ, and surface entropy d[γ·cos(θ)]/d*T* is currently an unanswered question.

It is the purpose of this article to explore the utility of eq 2 for nanoscale modeling and to analyze it in terms of its theoretical limits by utilizing the macroscopic properties of the intruding liquids. At the time of writing, this has not yet been experimentally demonstrated in the literature nor has the relationship between the surface entropy of liquids (solutions) and thermal output been explored. To achieve this, a nanoporous hydrophobic material was prepared and studied with multiple non-wetting solutions composed of pure water and ethanol, with each fluid possessing different surface entropy properties and by using scanning transitiometry as the accurate thermal measurement technique. This permits the validation of eq 2, with these measured thermal effects, generated in a confined nanoscale environment. The prospect that the process of reversible intrusion of non-wetting liquids (possessing a large macroscopic surface entropy) into nanoporous materials has the potential to compete with the current thermal energy storage (TES) technologies and offer complementary mechanisms of TES is created.

## Experimental Section

### Materials

The aqueous solutions of ethanol were prepared by mass on a top-loading balance into a clean 250 mL bottle. Water was distilled and degassed before mixing with absolute ethanol purchased from CHEMPUR (lot number 200-578-6), which was used as received without further purification. Solutions were degassed by sonication before preparing suspensions. Volume fractions were calculated using density data at 20 °C, provided by the NIST for water^37^ and ethanol.^37^ Ethanol was chosen as the solute for this study since it is non-toxic, and its aqueous solutions surface properties are found in the literature. It is easy to evaporate from the solid during reactivation processes.

Porous silica was purchased from DAVICIL as Grace 150A (Silica). The nominal pore size and specific surface area is 15 nm and 330 m^2^·g^–1^, according to the supplier. The pore size distribution was verified by transmission electron microscopy (TEM) and nitrogen adsorption techniques. The grafting of Silica was done following the procedure of Wong *et al*.^38^ A round-bottom flask was first charged with 16 mL of dry chloroform (Sigma-Aldrich, ≥99%) and purged with dry nitrogen for 30 min. A 0.4 g solution of Silica with an effective surface area of 264 m^2^·g^–1^ was then added into the flask under gentle stirring with a further nitrogen purge for 10 min. Subsequently, 0.2 mL of trichloro(1*H*,1*H*,2*H*,2*H*-perfluorooctyl)silane (Sigma-Aldrich, CAS #78560-45-9) was added into the flask. The reaction was then allowed to proceed at 25 °C at a stirring rate of 500 rpm for 48 h in an oil bath under dry nitrogen. The grafted silica was then washed in three cycles of dry chloroform (20 mL) and dried at 50 °C for 24 h. The grafting density of the materials is 4 μmol·m^–2^. This material is referred to as Silica-CF_3_ in the text. After the sample was characterized, the final material was shipped to each of the partner groups.

### Scanning Transitiometry (*PVT*-Calorimetry)

**Scheme 1:**
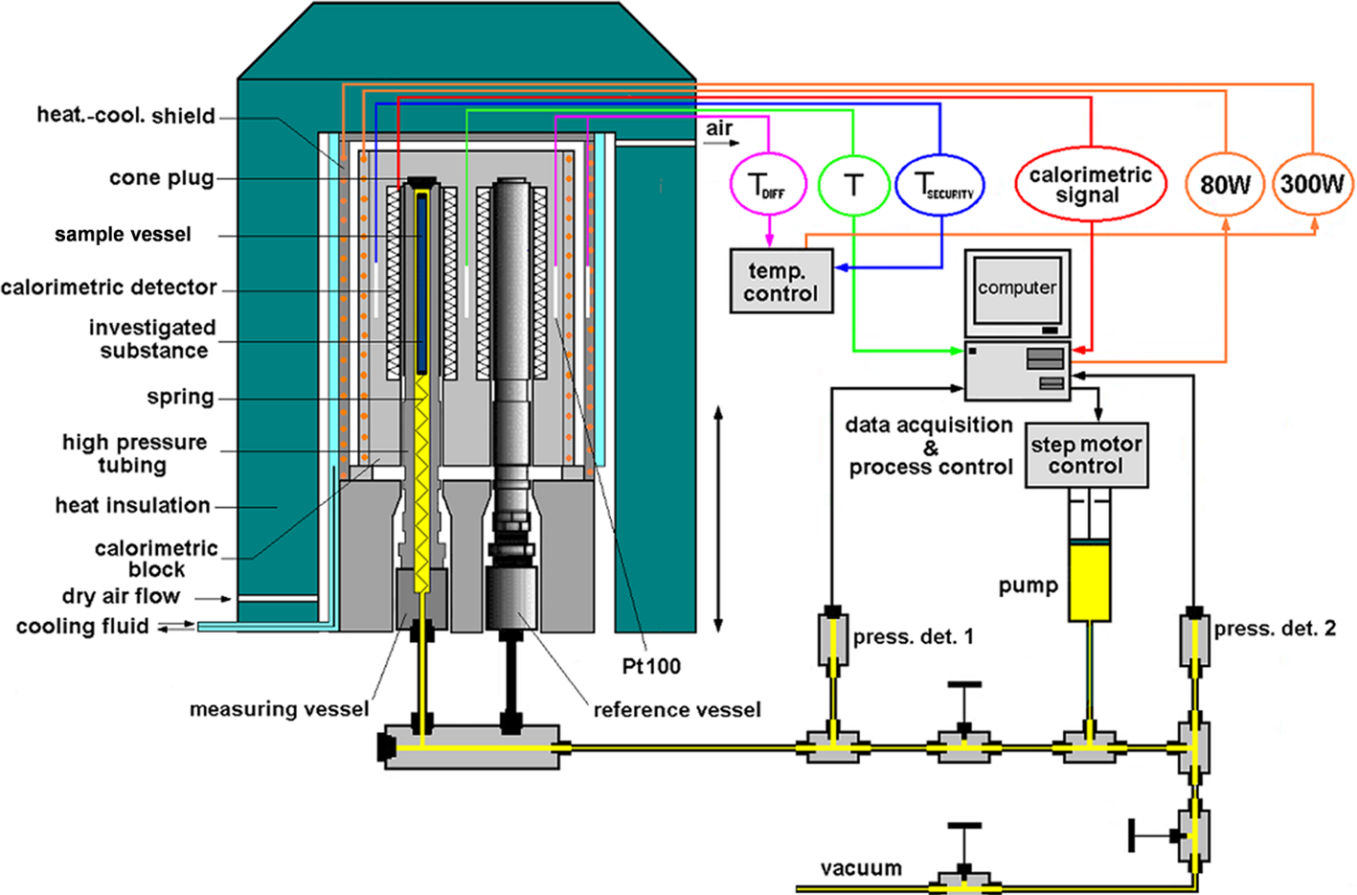
High-Pressure Scanning Transitiometer

A transitiometer from BGR-Tech was used to record the pressure–volume (*PV*) isotherms at 65 °C within the 0.1–45 MPa pressure range while simultaneously recording the associated thermal effects according to the procedures described by Grosu *et al*.^4,8^ using the equipment described by Chorążewski *et al*.^39,40^ located at the University of Silesia in Katowice, shown in Scheme 1.

A calorimeter cell rated for 200 MPa was attached to a manifold, which is connected to a high-pressure stepper motor. The suspension sample of Silica-CF_3_ and water was prepared as follows. The dry Silica-CF_3_ powder was weighed into a Teflon sample vessel with the dimensions of 7 cm in height, 6 mm in outer diameter, and 5 mm in inner diameter on an analytical balance. The recorded total mass of Silica-CF_3_ is 0.3463 ± 0.0005 g. The sample vessel was then packed and plugged with medical grade cotton. To remove the airspace within the filled Teflon vessel, it was placed in a 20 mL syringe barrel with degassed and distilled water. The syringe barrel piston was replaced, and excess air was expelled through the syringe barrel tip. The suspension within the Teflon vessel was created by first sealing the tip, and then the piston was pulled back to allow the air to evacuate from the Teflon vessel into the syringe barrel with water entering into the free space. This process of creating a vacuum was repeated three times. The same procedure was used to create the suspension with the other ethanol solutions. The remaining liquid was used to carefully fill the calorimeter cell and a spring inserted to hold the sample vessel in the proper position to record the heat effects. The calorimeter cell was closed using a torque wrench to prevent its over/under-tightening. *PV* compression-decompression cycles generated to record the heat effects were performed at scanning rate of 0.25 MPa·min^–1^ and then paused for 1.4 h at the maximum pressure of 30 MPa. Additionally, faster cycles of 1 MPa·min^–1^ were conducted without pauses. After each individual compression-decompression experiment, the Teflon vessel was placed into a glass-vacuum tube and gently heated at 90 °C with an oil bath while under vacuum to regenerate the Silica-CF_3_.

### *PVT*-Stand

A pressure–volume–temperature unit developed at the National Technical University of Ukraine “Igor Sikorsky Kyiv Polytechnic Institute” was used to ensure the repeatability of the observed effects and to perform preliminary experiments. The continuous isothermal *PV* compression-decompression cycles were conducted at significantly higher rates of 200 MPa·min^–1^. The equipment is described in the articles of Eroshenko^34^ and Fadeev.^29^

### Gas Adsorption

The surface properties were characterized in an automated gas adsorption analyzer (Micromeritics ASAP 2460). Isothermal nitrogen sorption curves of the samples were measured after outgassing at 200 °C under vacuum conditions for 5 h. The multipoint surface area was evaluated with the Brunauer–Emmett–Teller method over the pressure range of *P*/*P*_0_ = 0.075–0.35, where *P*_0_ is the saturated pressure of nitrogen. The pore size distribution was obtained using the Barrett–Joyner–Halenda model, which is fitted to the desorption isotherm branch. The total pore volume was determined from the volume adsorbed at *P*/*P*_0_ = 0.98. The surface area was calculated to be 264 m^2^·g^–1^ for Silica and 100 m^2^·g^–1^ for Silica-CF_3_. Once characterized, the final material was shipped to each of the partner groups.

### Transmission Electron Microscopy

TEM measurements were performed using an FEI Tecnai F20 electron microscope operating at 200 kV. For TEM measurements, samples were dispersed in ethanol and sonicated. The solution was then transferred onto a holey carbon film fixed on a 3 mm copper grid (200 mesh).

### Contact Angle Measurements

A Krüss Drop Shape Analyzer (DSA 100) was used to measure the contact angles of water on the flat surface of the Silica-CF_3_ powder. The powder was placed in a watch glass, and the surface was smoothed with a clean glass surface. The smoothed powder surface was inspected for large voids and shallow slopes, which would allow the water drops to roll and were smoothed out. The sample in the watch glass was put into the sample temperature control chamber and given 15 min to reach thermal equilibrium. The drops of water were then gently rested on the surface. Ten independent measurements were performed with multiple water drops of which the average values are presented. The precision of these measurements is about ±1° with respect to the machine optics. The largest standard uncertainty between the contact angles of multiple liquid drops is ±4°. The tangent 1 mode included with the drop shape analysis software provided by Krüss was used to measure the contact angles of water at the temperatures of 25, 45, and 65 °C. The contact angles of powdered ZIF-8 were done using the same model of apparatus but using upgraded software with the Young–Laplace method.

## Results and Discussion

**Figure 1:**
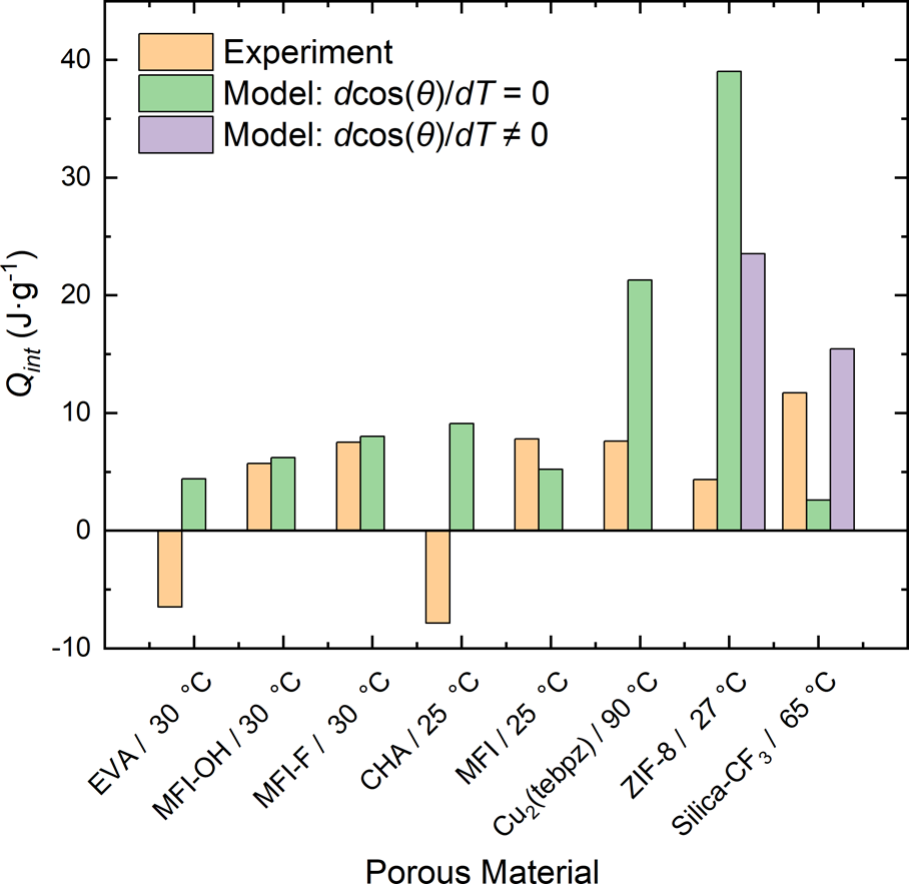
Measured heat of intrusion of water into ZIF-8,^9^ EVA,^5^ MFI-OH,^5^ MFI-F,^5^ CHA, MFI zeolites,^2^ Cu_2_(tebpz) MOFs,^4^ and Silica-CF_3_ (this work), compared to calculated values according to the model (eq 2) considering d cos(θ)/d*T* = 0 and d cos(θ)/*dT* ≠ 0.

To begin, the authors propose to evaluate the current predictions of eq 2 using the currentily available calorimetric data for porous materials and water as the non-wetting liquid. At the time of writing, there have been seven separate systems investigated with various results reported. Figure 1 demonstrates these experimental results combined with the surface entropy of water calculated from the IAPWS curated data^41^ and estimated contact angles taken from their respective articles if present.

For the zeolite systems studied by Coffiard *et al.*,^5^ they show that the first general approximation of the temperature-independent contact angle, (dγ/d*T*)·(cos θ)*,* for systems composed of H_2_O and MFI-OH (experimental 5.7 J·g^–1^/calculated 6.2 J·g^–1^) or H_2_O and MFI-F (experimental 7.5 J·g^–1^/calculated 8.0 J·g^–1^) are consistently 0.5 J·g^–1^ higher. In comparison, Karbowiak *et al.*(2), MFI showed a higher thermal energy compared to the model. For the calculation, the specific surface area is assumed to be the same as MFI-OH,^5^ but variations in the synthesis may lead to different surface area values, which were not reported in the original article.^2^ Overall, the predicted thermal energies move in the same direction and have an appropriate magnitude. When the pore size is much larger or posseses a different geometry, which is shown for EVA^5^ and chabazite zeolite,^2^ the thermal effect of intrusion is exothermic.^2,6^ For MOFs like ZIF-8 and Cu_2_(tebpz), the thermal effect is consistently overpredicted, suggesting that the contact angle’s temperature dependence may play a defining role in the overall heat generation. Additionally, the intrusion of guest molecules into MOFs, and ZIF-8 in particular, is often coupled with flexibility effects, such as “opening the gate”.^42^ This may introduce additional not yet unaccountable effects between the used model and experimental data. For the last two experiments (ZIF-8 and Silica-CF_3_, in Figure 1), their temperature-dependent contact angle effects were applied. The inclusion of this property brings the thermal energy prediction closer to those measured values.

In summary, it can be argued that the agreement between the model and experiment is acceptable for ridged microporous zeolites with an MFI type of topology as well as for grafted silicas when the temperature-dependent contact angle is applied. What is clearly seen from Figure 1 is that eq 2 with the temperature-independent contact angle fails to predict the exothermic heat of intrusion, which was reported for chabazite zeolite^2^ and EVA.^5^

**Figure 2:**
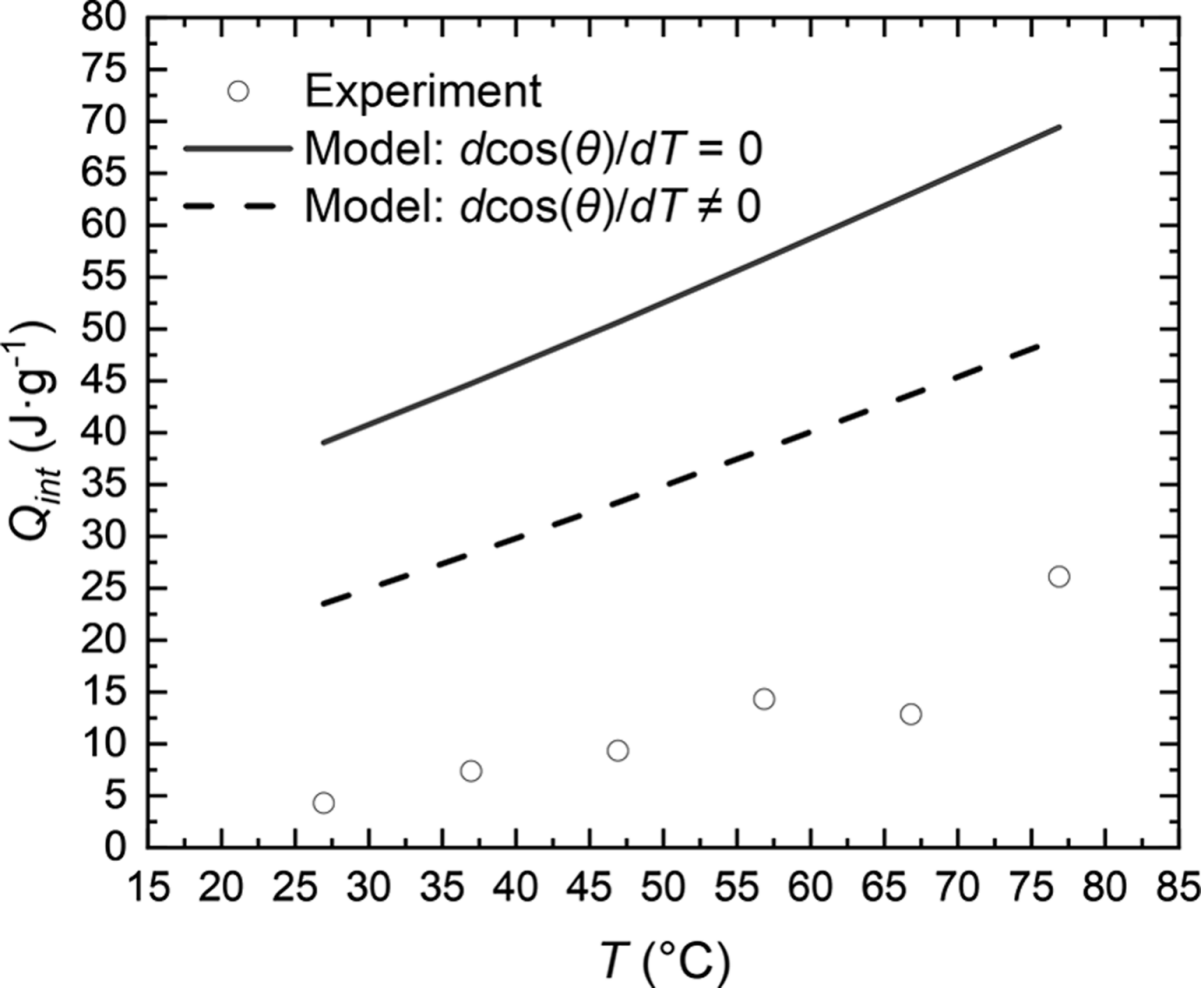
Measured heat of liquid intrusion of water into ZIF-8 (empty circles) from Grosu *et al.*(9) and calculated values according to eq 2 considering d cos(θ)/d*T* = 0 (black solid line) and d cos(θ)/d*T* ≠ 0 (black dashed line).

While using eq 2, it must be understood that, for mesoporous materials, it is possible to identify and determine the contact angle, but for microporous materials, the pore opening is below 1 nm in diameter, and the contact angle does not exist in this situation. However, should the surface be chemically similar to the microporous material, the macroscopic contact angle of liquid (including its temperature dependence) on this surface would contain information on the molecular interaction between the liquid and solid. In macroscopic terms, this interaction determines the sign and magnitude of heat related to spreading a liquid over the surface. It is necessary to understand whether this macroscopic information on liquid spreading over the surface can be useful for the case of liquid intrusion into the microporous material. This was evaluated by measuring the contact angle of water on the smoothed surface of ZIF-8, which possess dθ/d*T* = 0.0006 K^–1^ over the measured temperature range of 30–60 °C (see Table S1 in the Supporting Information). This contact angle behavior is not uncommon, with Laouir *et al*.^33^ demonstrating that this behavior with water on silicone-coated glass and water on hexatriacontane. When the temperature dependence of contact angle is considered, the heat of intrusion is still overestimated by the model, but the agreement between the experiment and model improves as seen in Figure 2 (and Figure 1)*.* This movement toward the experimental value is expected when considering the analysis by Laouir *et al*.^33^ and Borman *et al.*(36) regarding these properties.

This is a valuable result, which suggests that, with further study of microconfinement and the introduction of corresponding corrections, it may be possible to predict the heat of intrusion into micropores based on macroscopic values, such as surface entropy and the temperature-dependent contact angle. Currently, the used macroscopic approach is more applicable for mesoporous materials but, considering that the contact angle is defined by the surface interactions between a solid and liquid, this attempt to understand whether the macroscopic contact angle (large drop of liquid) on the surface of the porous material of interest (a smoothed surface of ZIF-8) and its temperature dependence can help to predict the heat of intrusion (or at least its thermal sign).

**Figure 3:**
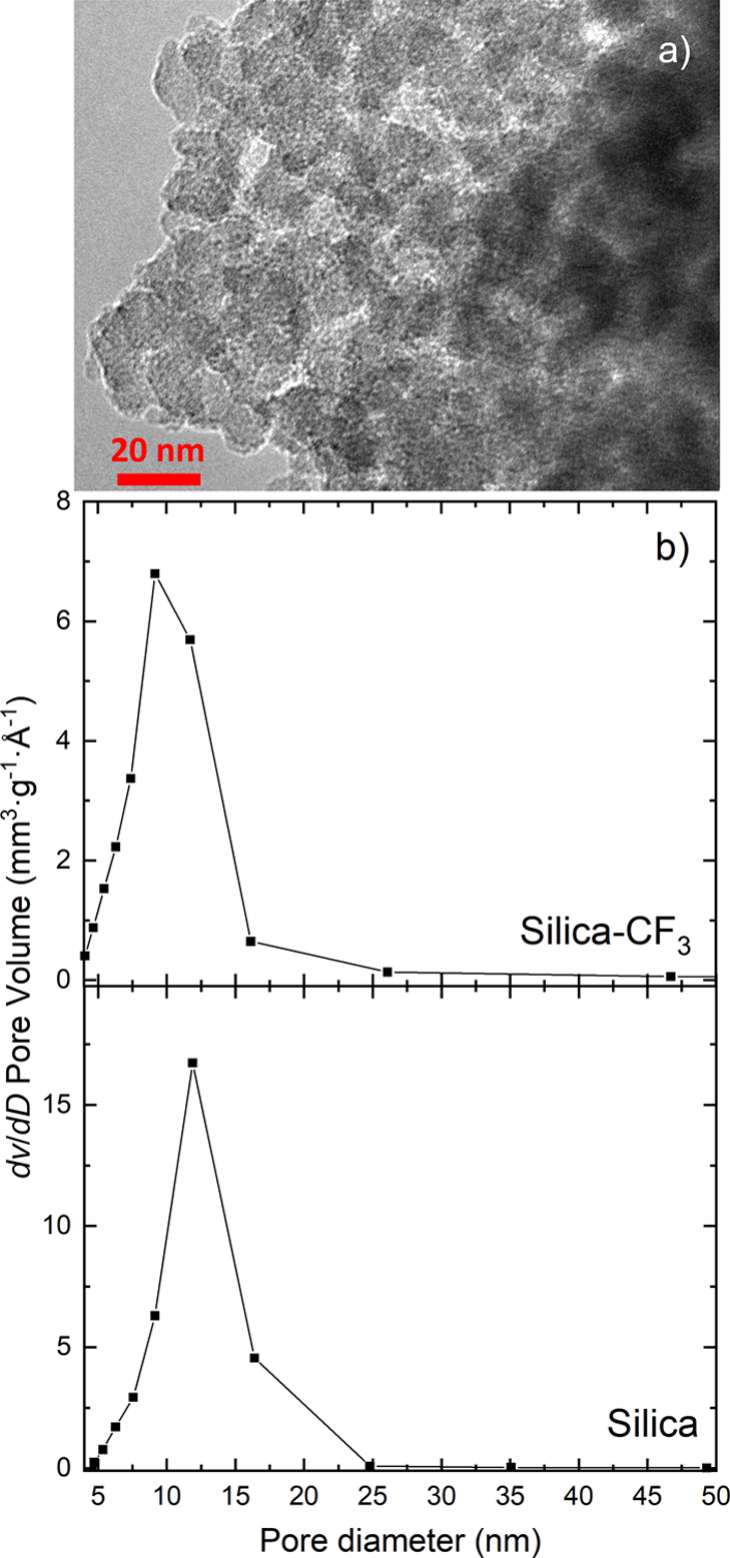
(a) TEM micrograph of Silica and (b) pore size distribution of Silica and Silica-CF_3_.

To further explore this relationship, a new mesoporous material Silica-CF_3_ was prepared. The TEM characterization of the Silica (pre-graft) shows the disordered spherical pores with a diameter of approximately 15 nm as seen in Figure 3a. This result is supported with nitrogen adsorption experiments for both Silica and Silica-CF_3_ (Figure 3b). The addition of hydrophobic grafting reduces the pore diameter to 10 nm and the surface area to 100.7 m^2^·g^–1^.

**Figure 4:**
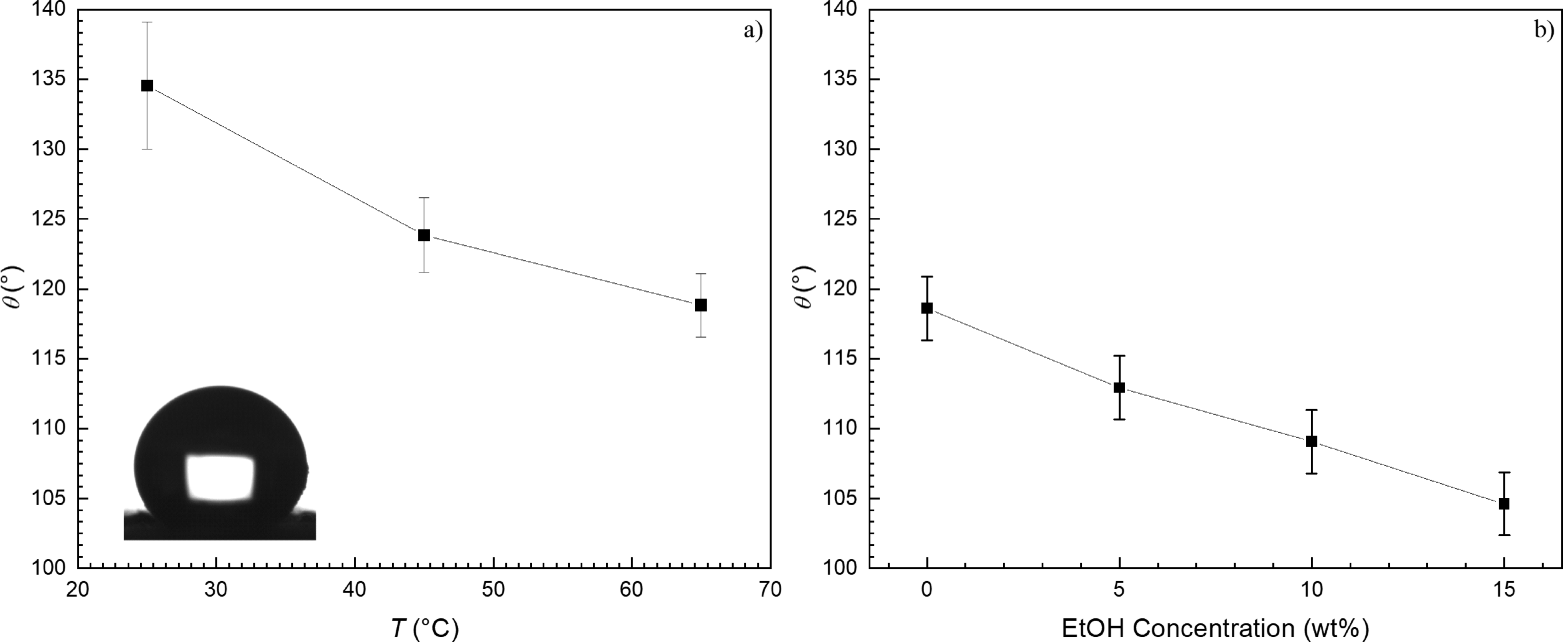
Contact angle (°) on the surface of Silica-CF_3_ for (a) water and (b) aqueous ethanol solutions at 65 °C using θ_E_ = φ · dθ/dφ + θ_H_2_O_.

The contact angle measurements in Figure 4a show the hydrophobic nature of Silica-CF_3_ at temperatures of 25, 45, and 65 °C. These results show that the contact angle displays a negative temperature dependence, which is evaluated to be dθ/d*T* = −0.0063 K^–1^. This behavior is consistent with the temperature-dependent behavior for the surface tension of water.^41^ Accurate measurements of aqueous ethanol solutions at 65 °C were not possible to obtain due to the increasing vapor pressure of the drop with increasing temperature. To substitute, the contact angles of the ethanol solutions, *θ*_E_, were estimated using the linear dependence of the ethanol concentration by volume percent φ. The following equation was used: θ_E_ = φ · dθ/dφ + θ_H_2_O_, where θ_H_2_O_ is the measured contact angle of pure water on the surface of Silica-CF_3_ at 65 °C and dθ/dφ = – 0.0133 (θ in radians) according to the smooth surface data of Spencer *et al*.^43^ The contact angle dependence on ethanol concentration at 65 °C is demonstrated in Figure 4b.

**Figure 5:**
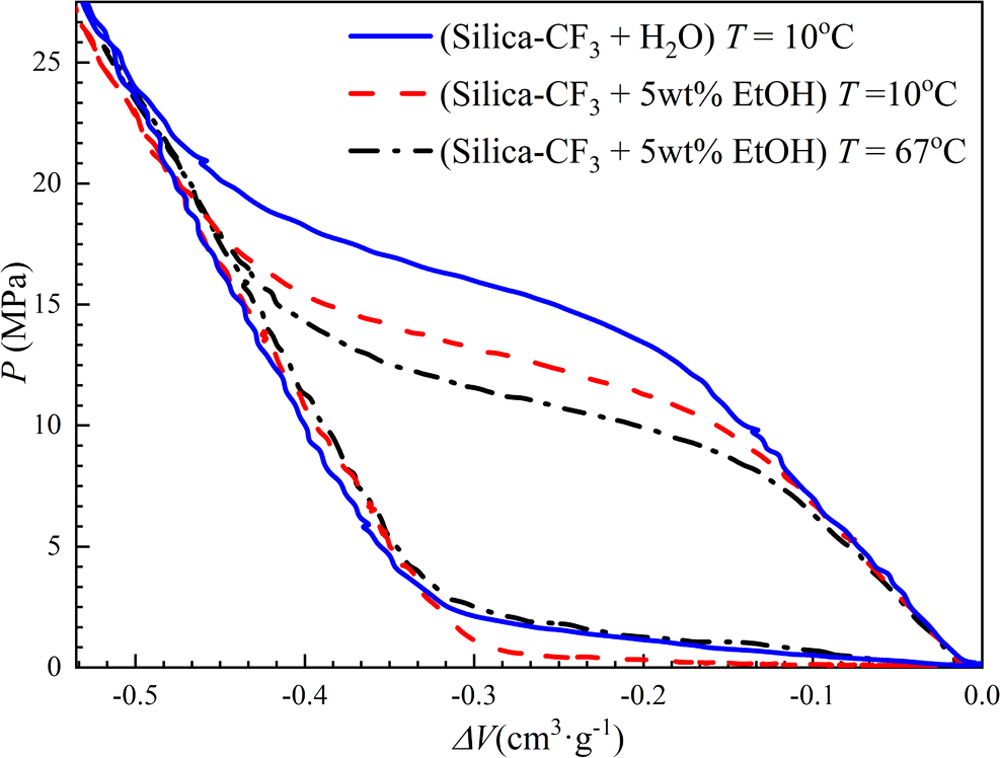
*PV* isotherms of (Silica-CF_3_ + H_2_O/5 wt % EtOH solution) systems at *T* = 10 or 67 °C at a cycling rate of 200 MPa·s^–1^.

The *PV* isotherms of Silica-CF_3_ with water and a 5 wt % ethanol (EtOH) solution were measured using the *PVT*-stand for three rapid consecutive loops to record both the liquid intrusion and extrusion at a rate of 200 MPa·min^–1^. These results are reported at 10 °C (water and 5 wt % EtOH) and 67 °C (5 wt % EtOH) and are displayed in Figure 5.

The observed hydrophobic nature of Silica-CF_3_ requires a high pressure to drive the intrusion, which is observed by the appearance of a plateau on the *PV* isotherm for water and 5 wt % ethanol solutions at the intrusion pressure (*P*_int_).

The addition of ethanol to water consequently decreases both surface tension^44^ and contact angle,^43^ which results in the decrease in *P*_int_ according to eq 1. This is seen in the corresponding *PV* isotherms in Figure 5, collected by rapid cycling. The *P*_int_ of 5 wt % EtOH solution is approximately 3 MPa lower, compared to the *P*_int_ of pure water at 10 °C. Increasing the temperature to 67 °C causes the surface tension of the ethanol solution to decrease, leading to the observed decrease in *P*_int_. The complete extrusion of liquid takes place upon the consecutive rapid*,* decompression of the system with repeated compression-decompression cycles showing the same reproducible behavior to the first cycle. In general, the tested fluids showed the same large *PV* hysteresis, which describes the material as a molecular shock absorber or bumper.^13−18^ At 10 °C, water extrudes from the pore at a pressure of 1 MPa, while the ethanol solution extrudes at 0.2 MPa. Increasing the temperature of the experiment to 67 °C also increases the extrusion pressure of the ethanol solution to 1 MPa.

For the thermal effects, a separate batch sample was measured using scanning transitiometry. The compression experiments were performed at a much slower rate of 0.25 MPa·min^–1^ and then paused for 1.4 h at the maximum pressure of 30 MPa. Upon performing the decompression phase of the experiment, no extrusion was observed. When the pause step was omitted and the scanning rate increased to 1 MPa·min^–1^, liquid extrusion was still not observed. These results are consistent with the observation of Qiao *et al*.,^45^ where it was demonstrated that complete dissolution of gas molecules in a liquid under high pressures may inhibit or prevent (in this case) the extrusion process.

**Figure 6:**
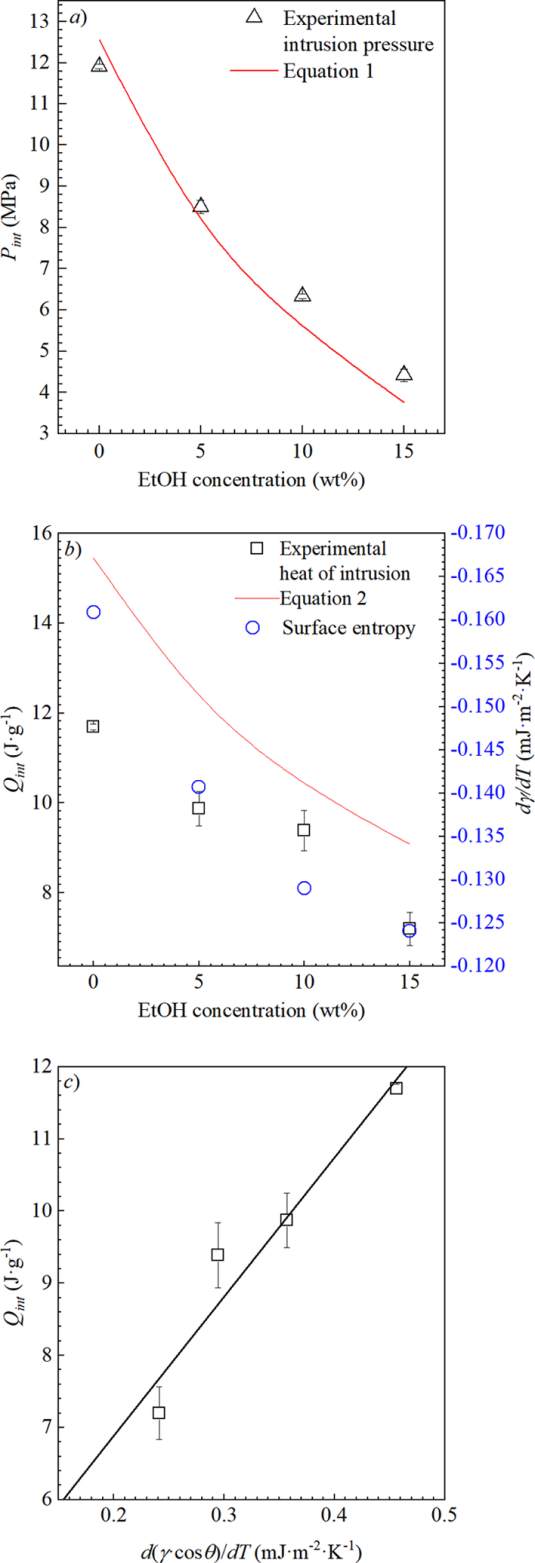
(a) Intrusion pressure, *P*_int_, (b) heat of intrusion, *Q*_int_, and (c) correlation between the heat of intrusion and surface entropy (black line).

Figure 6 shows the simultaneously collected *P*_int_ and *Q*_int_ data of liquid intrusion into the pores of the solid by scanning transitiometry. The results are the averages and standard deviation of three independent experiments. In Figure 6a, the results of the *PV* experiments are presented with the values from eq 1, which was found to predict the *P*_int_ reasonably well. This indicates that the macroscopic parameters of γ and θ are sufficient to describe the mechanical intrusion process for a 10 nm pore.

To calculate the modeled values intrusion pressure in thermal response, as seen in Figure 6, the γ and dγ/d*T* values are taken from the experimental data from Vazquez *et al*.^44^

In Figure 6b, the endothermal heats are slightly scattered compared to the *P*_int_ data but the overall trend of decreasing *Q*_int_ and *P*_int_ is consistent. This is primarily directed by the dγ/d*T* of water and increasing the concentration of ethanol in solution. Equation 2 predicts that the amount of thermal energy is 20% greater than the experimental data. The consistency of this gap between predicted and measured values is very encouraging since it shows that the dγ/d*T* of a liquid holds significant consequences for thermal energy storage capacity. This estimation includes the d(cos θ)/d*T* of water*,* as shown in Figure 1, and when absent, the predicted thermal energies are 85% lower, indicating that the magnitude of thermal energy is dependent on contact angle properties. This relationship is reinforced in Figure 6c, which highlights the direct linear relationship between the d[γ·cos(θ)]/d*T* and thermal effects of intrusion, *Q*_int_. This emphasizes that these macroscopic quantities can be used to predict the thermal effects at the nanoscale level.

Again, using this simplified macroscopic approach, it is assumed that the liquid is interacting with a smooth, ideal macroscopic surface. This demonstrates that this macroscopic approach does produce a reasonable value for the *Q*_int_ of liquid intrusion into a nanoporous material. This includes tailoring the combinations based on the known properties of the liquid (solution) and mesoporous solid to suit the application. For example, specific surface areas of MCM-41 with superhydrophobic^46^ and hydrophobic^47^ grafting may range within 801 m^2^·g^–1^ and 1307 m^2^·g^–1^. Using the same surface entropy and contact angle properties presented here, these systems’ values may produce an estimated 119–194 J·g^–1^ of thermal energy stored.

The limits of the model do require improvement to comprehend what is occurring in the pores of the sample, which is beyond the scope of this paper. The thermal effects related to the the pore geometry and variation topology have previously been investigated using simulations and experiments. At this point, only ideal approximations have been considered and include that the entire system has been filled with liquid, which is similar to the spreading of liquid across a surface. As well, there is also the consideration from Borman *et al*.^36^ about the formation and destruction of the meniscus inside the pore and the process of pore filling. In an amorphous system, the pore system would be difficult to model since the pores can be of different sizes and geometries. This explains why the model consistently estimates a higher energy than measured by the transitiometer and is more challenging to model.

**Scheme 2:**
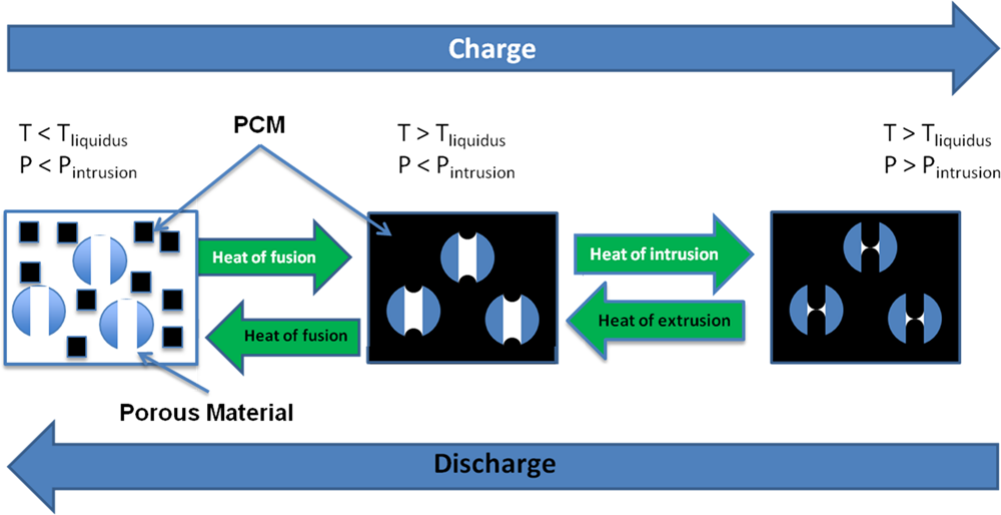
Thermal Energy Storage Using Melting-Intrusion-Extrusion-Solidification Cycles

The energy capacity of these non-wetting systems can be improved by creating a hybrid system comprising a PCM for the non-wetting liquid and subjecting the system to melting-intrusion-extrusion-solidification cycles under isochoric conditions (Scheme 2).

The TES device’s mechanism will function based on an expanding fluid, driven by the accumulation of thermal energy in an isolated isochoric system. The example given in Scheme 2 demonstrates a phase change material (PCM), which first transforms from a solid to a liquid (T_liquidus_) by the accumulation of thermal energy of phase transition. As the thermal energy continues to accumulate, the liquid expands, and the system pressurizes. When the accumulated pressure increases above the *P*_int_, the liquid will enter into the pore and thus stores the thermal energy as the heat of intrusion. Since the temperature is increasing, the surface tension will decrease accordingly and also lower the intrusion pressure. The reverse process (cooling) discharges the accumulated thermal energy as a sum of the heat of extrusion, heat of fusion, and sensible heat of cooling.

## Conclusions

The use of macroscopic properties, which include the surface entropy (d[γ·cos(θ)]/d*T*) of non-wetting liquids and the specific surface area (Ω) of a mesoporous solid was used to evaluate the model to predict the thermal effect (heat) of liquid intrusion into Silica-CF_3_ and highlights the potential significance of the temperature-dependent contact angle for the prediction of its thermal energy values. Meanwhile, the liquid intrusion into flexible microporous metal–organic frameworks requires further improvement.

Potentially by using materials with the largest values for surface entropy and specific surface area, the intrusion of non-wetting liquid into nanopores can be used for thermal energy storage. The energy capacity can be further increased by partnering it with a phase change material as the non-wetting liquid and taking advantage of the melting-intrusion-extrusion-solidification cycle.

## Supporting Information Available

(SI Table 1) Contact angle measurement of water drops on ZIF-8 (MOF) and (SI Table 2) contact angle measurements of a water drop on Silica-CF_3_ (PDF)
