# Supplementary material for: The Effect of Surface Entropy on the Heat of Non-Wetting Liquid Intrusion into Nanopores
Source: Langmuir. 2021 Apr 12;37(16):4827–35. doi: 10.1021/acs.langmuir.1c00005 (PMC8154867; doi:10.1021/acs.langmuir.1c00005)
Supplement: Supplementary file 1 — la1c00005_si_001.pdf [file la1c00005_si_001.pdf]

Supporting Information File:

The Effect of Surface Entropy on the Heat of Non-Wetting Liquid Intrusion into Nanopores

Alexander R. Lowe,[1] William S. Y.Wong,[2] Nikolay Tsyrin,[3] Mirosław A. Chorążewski,*[1] Abdelali Zaki,[5] Monika Geppert-Rybczynska,[1] Victor Stoudenets,[3] Antonio Tricoli,[2,4] Faik Abdessamad, [5,6] Yaroslav Grosu,[5,6]**

1 Institute of Chemistry, University of Silesia, Szkolna 9, 40-006 Katowice, Poland

2 Nanotechnology Research Laboratory, College of Engineering and Computer Science, The Australian National University, Canberra ACT 2601, Australia

3 Laboratory of Thermomolecular Energetics, National Technical University of Ukraine "Igor Sikorsky Kyiv Polytechnic Institute", Pr. Peremogy 37, 03056 Kyiv, Ukraine

4 Nanotechnology Research Laboratory, University of Sydney, 2006 NSW, Australia

5 Centre for Cooperative Research on Alternative Energies (CIC energiGUNE),Basque Research and Technology Alliance (BRTA), Alava Technology Park, Albert Einstein 48, 01510 Vitoria-Gasteiz, Spain

6 Materials Science, Energy and Nano-engineering Department, University Mohammed VI Polytechnic, Lot 660, Hay Moulay Rachid, 43150 Ben Guerir, Morocco

SI Table 1: Contact angle measurement of water drops on ZIF-8 (MOF) in degrees (°) and radians (unitless).

Temperature	Contact Angle (θ)		± σ (Contact Angle)	
°C	°	radian	°	radian
30.4	160.0	2.79	4.8	0.08
40.8	160.5	2.80	4.1	0.07
51.3	160.0	2.79	4.2	0.07
60.3	158.1	2.81	2.4	0.04
Y =m(K^{-1}) x + b(unitless)	0.0006	± 0.0003	2.773	± 0.018

SI Table 2: Contact angle measurements of a water drop on Silica-CF_3 in degrees (°) and radians (unitless).

Temperature	Contact Angle (θ)		± σ (Contact Angle)	
°C	°	radian	°	radian
25	134.5	2.35	4.5	0.08
45	123.9	2.16	2.7	0.05
65	118.8	2.07	2.3	0.04
Y =m(K^{-1}) x + b(unitless)	-0.0063	±0.001	2.47	±0.08
